# ResNet incorporating the fusion data of RGB & hyperspectral images improves classification accuracy of vegetable soybean freshness

**DOI:** 10.1038/s41598-024-51668-6

**Published:** 2024-01-31

**Authors:** Yuanpeng Bu, Jinxuan Hu, Cheng Chen, Songhang Bai, Zuohui Chen, Tianyu Hu, Guwen Zhang, Na Liu, Chang Cai, Yuhao Li, Qi Xuan, Ye Wang, Zhongjing Su, Yun Xiang, Yaming Gong

**Affiliations:** 1 Institute of Vegetables, Key Laboratory of Vegetable Legumes Germplasm Enhancement and Southern China of the Ministry of Agriculture and Rural Affairs, Zhejiang Academy of Agricultural Sciences, Hangzhou, China; 2 Institute of Cyberspace Security, Zhejiang University of Technology, Hangzhou, China; 3 Zhejiang Yuncheng Information technology Co Ltd., Hangzhou, China; 4 Faculty of Engineering, Lishui University, Lishui, China

**Keywords:** High-throughput screening, Classification and taxonomy, Machine learning, Plant physiology

## Abstract

The freshness of vegetable soybean (VS) is an important indicator for quality evaluation. Currently, deep learning-based image recognition technology provides a fast, efficient, and low-cost method for analyzing the freshness of food. The RGB (red, green, and blue) image recognition technology is widely used in the study of food appearance evaluation. In addition, the hyperspectral image has outstanding performance in predicting the nutrient content of samples. However, there are few reports on the research of classification models based on the fusion data of these two sources of images. We collected RGB and hyperspectral images at four different storage times of VS. The ENVI software was adopted to extract the hyperspectral information, and the RGB images were reconstructed based on the downsampling technology. Then, the one-dimensional hyperspectral data was transformed into a two-dimensional space, which allows it to be overlaid and concatenated with the RGB image data in the channel direction, thereby generating fused data. Compared with four commonly used machine learning models, the deep learning model ResNet18 has higher classification accuracy and computational efficiency. Based on the above results, a novel classification model named ResNet-R &H, which is based on the residual networks (ResNet) structure and incorporates the fusion data of RGB and hyperspectral images, was proposed. The ResNet-R &H can achieve a testing accuracy of 97.6%, which demonstrates a significant enhancement of 4.0% and 7.2% compared to the distinct utilization of hyperspectral data and RGB data, respectively. Overall, this research is significant in providing a unique, efficient, and more accurate classification approach in evaluating the freshness of vegetable soybean. The method proposed in this study can provide a theoretical reference for classifying the freshness of fruits and vegetables to improve classification accuracy and reduce human error and variability.

## Introduction

Vegetable soybean (VS) is typically harvested immaturely when the seeds have filled  90% of the seed cavity and the pod color has not yet turned yellow^1,2^. The premature harvest makes VS rich in free amino acids and carbohydrates, a fresh green color, and a soft and sticky texture; all of which are important organoleptic quality properties of VS^3^. However, the texture and physicochemical properties of immature VS still undergo complex changes after harvest due to continuing metabolic processes. These changes lead to a rapid decrease in freshness, such as yellowing, increased spots, and decreased sweetness and flavor, which can significantly affect the nutritional value, taste, and appearance quality of VS^4^. It is reported that the sucrose contents can be drastically decreased over 60%, from 8.7%–10.4% to 3.0%–3.1%, within one day stored in ${25}^{\circ }\hbox {C}$^5^.

The freshness of VS plays a crucial role in attracting consumers^6^. Various factors, such as genotype, pod mature degree at harvest, storage time and conditions, can affect the physicochemical properties, thereby influencing the freshness of VS^2,4,7^. Researchers have examined that the physicochemical characteristics, including total soluble sugar, moisture, total free amino acid, starch, protein, oil, pod green intensity, and seed hardness, are closely connected to the taste and appearance quality of VS^8–12^. The color of the pod is the first aspect of the appearance quality that consumers pay attention to. Additionally, in sensory quality evaluation, the total soluble sugar is reported significantly positively correlated with the taste-quality score (*r* = 0.864, *p* < 0.01)^13^. The tender texture or low hardness is one of the important characteristics of VS for better taste and easier processing. VS with low moisture content have higher hardness and are scored lower in overall sensory evaluation^14^. The content of free amino acids significantly affected the umami taste and flavor of vegetable soybeans^10^. In the agricultural industry standard of the People’s Republic of China, “Vegetable Soybean Varieties Quality” (NY/T3705-2020), the aforementioned eight physicochemical parameters serve as the basis for quality assessment.

According to our knowledge, research is scarce on how to identify and evaluate the freshness of VS. Traditionally, freshness is quantified through sensor evaluation and physical and chemical index testing. However, sensory evaluation is subjective and lacks accuracy and universality. It is performed by individual experts and the results all depend on their sight, touch, taste, and smell. The measurement and evaluation of freshness-related indicators through physical and chemical experiments are the most common method. However, these processes are very time-consuming and laborious. As one of the most efficient techniques for plant nutrients analysis^15^, proton nuclear magnetic resonance spectroscopy has been used to determine sugars, organic acids, and amino acids changes of VS seeds^2^. Although this method does not require complex chemical reactions, the contribution of each indicator to freshness is challenging to define accurately. Therefore, it is important and urgent to develop an objective, efficient, and comprehensive evaluation technology for VS freshness research.

Non-destructive analysis of food freshness and quality using optical sensing technology has become a current research hotspot^16–18^. In its early phase, this technique was primarily utilized for detecting fungi which is responsible for rotting citrus fruits^19^, identifying mechanical damage in mangoes, categorizing agricultural produce, and assessing the ripeness of tomatoes^16,20^. Thereinto, hyperspectral imaging provides both spatial and spectral information, making it ideal for monitoring the ripening process of agricultural products, such as ethylene biosynthesis, chlorophyll degradation, nutrient conversion and respiratory action^21^. Machine learning classification algorithms based on RGB images have been widely used in the quality grading of agricultural products^22,23^, as well as ripeness monitoring of bell peppers^24^ and gooseberries^25^. The deep residual networks (ResNet) can greatly enhance the efficiency of neural networks in the image classification tasks^26^. Convolutional Neural Network (CNN) is a kind of Feedforward Neural Network with a deep structure including convolution calculation. It is one of the representative algorithms of deep learning. At present, CNN technology has been used for fruit and vegetable classification and fruit ripened detection based on RGB images^27,28^. Recently, a deep learning system for multi-category classification is proposed based on an improved YOLOv4 model^29^. The system first identifies the type of objects in the RGB image, and then categorizes them as fresh or rotten. However, in their work, only two categories are distinguished with limited accuracy.

The RGB image recognition^30^ technology is widely used in the study of food appearance evaluation. In addition, the hyperspectral image has outstanding performance in predicting the nutrient content of samples. However, research on classification models based on the fusion data of RGB and hyperspectral images are scarce. Therefore, in this work, we classified the freshness of VS by different stored times and determine their physicochemical properties. Then the RGB and hyperspectral images were collected in chronological order. ResNet is a widely used deep neural network architecture and is excellent in tasks such as image classification and target detection. It also has good generalization capabilities across different datasets and tasks. ResNet makes it easier to train deep networks while mitigating vanishing gradients. Hyperspectral images and RGB images cannot be directly fused because their dimensions are 1D single-channel and 2D dual-channel, respectively. In that case, the ResNet-based model is helpful in capturing their complex relationships. Moreover, the residual connection of ResNet can fully consider the correlation between multi-channel data. Therefore, ResNet model is capable of integrating these two types of data efficiently. Based on the fusion of RGB and hyperspectral image data, we develop a vegetable soybean freshness classification model named ResNet-R &H (RGB & Hyperspectral imagery).

## Materials and methods

In this section, we present the sample management method, including physical and chemical characterization analysis, RGB and hyperspectral image acquisition, calibration, and processing. For vegetable soybean freshness classification, we develop ResNet-R &H model, which is based on the fusion of RGB image and hyperspectral data.

### Experimental materials

The genotype “Zhenong 6”, one of China’s most representative and popular VS cultivars^31^, is used. The experimental materials were planted in the experimental field of the Zhejiang Academy of Agricultural Sciences on April 5, 2022. One thousand well-developed and disease-free three seed pods were harvested at the R6 stage, when the seeds were filled  90% of the seed cavity, and the pods and seeds were bright green in color. The pods are stored in a controlled environment greenhouse, maintaining a constant temperature of ${24}^\circ \hbox {C}$ , an ambient humidity of 60%, a CO_2_ concentration of 400 ppm, a 12/12-hour light/dark photoperiod, and a photosynthetic flux density of 600 μ mol m^−2^s^−1^. The harvested pods are divided into two groups: one consisting of 100 pods, and the other consisting of 900 pods. On the 1st, 3rd, 5th, and 7th days, the 100 pods from the first group are numbered and subjected to collect RGB and hyperspectral images. Simultaneously, 200 pods are randomly selected from the second group, and their seeds are extracted to perform quantitative measurements of hardness, soluble sugar, free amino acid, starch, moisture, protein, and oil content. The collection of plant materials complied with relevant institutional, national, and international guidelines and legislation.

### PCI extraction

#### Physical characterization

The TA.XT Plus texture analyzer (Stable Micro Systems Ltd., UK) was adopted to test the seed hardness of VS according to the method described by our previous report^14^. Put it briefly, a cylinder stainless probe with a diameter of ${2} \hbox {mm}$ was equipped for puncture testing. The puncture test speed is ${1} \hbox {mm s}^{-1}$ and the test time (*t*) is 2 s. The averaged mechanical work (calculated as $W=\int _0^tf(t)dt$) from six parallel tests was used as the seed hardness index.

#### Chemical compositions

To measure the moisture content, twenty green seeds were heated in a constant temperature oven at 75$^\circ \hbox {C}$ until the weight stops dropping. The moisture content of the fresh VS seeds was then determined gravimetrically^32^. Freeze-dried VS seeds were ground into powder for determination of amino acid content, soluble sugar, protein, crude protein, and oil. The analysis of free amino acids was performed using a Hitachi 8900 amino acid analyzer (Hitachi High-Technologies, Tokyo, Japan) referring to the literature^3^. The soluble sugar content was determined by anthrone colorimetry using glucose as the standard ^33^. The Kjeltec TM2300 autosampler system (Foss Analytical, Hillerd, Denmark) was adopted to measure protein content. The crude protein was estimated using a conversion factor of 6.25^11^. Oil content is estimated using a Soxtec 2050 Soxhlet extraction system (Foss Analytical, Hillerd, Denmark)^34^. Each sample was analyzed three times to ensure accuracy.

### Image data processing

#### Image acquisition

RGB images of VS pods were captured using a Canon EOS 200D II camera in a RGB image acquisition system. The camera parameters were as follows: lens f=18–55mm, focal length 0.25 m, shutter speed 1/4000 to 1 sec, aperture f/4−5.6, lens mounting height 46mm. The digital images were converted into RGB (red–green–blue) and input to the CIELAB system using the Conversion Munsell (program version 4.01), thus deriving the parameters of the Lab color model. To assess the green intensity of VS, the hue parameter [H = arctang (b/a)] was calculated ^35–37^.

A hyperspectral image acquisition system^38^ was adopted to collect hyperspectral images of VS. The system consisted of a Pika XC hyperspectral camera, an imaging spectrograph, a high-performance Schneider Xenoplan 1.4/17 lens unit, four 15-W 12-V tungsten halogen lamps, and a Spectral Image data acquisition software. In this study, reflectance data was obtained within a spectral range spanning 386 to 1004 nm. The spectral resolution employed for this data collection was set at 1.3 nm. To initiate the spectral analysis process, the spectrometer undergoes a preheating phase aimed at achieving a consistent light irradiation. Subsequent to this step, a black and white correction procedure was executed. The initial stage of this correction process involves utilizing a whiteboard as the background. Following the acquisition of the background image of the whiteboard, the whiteboard was removed and then replaced by the samples, thereby allowing for the capture of hyperspectral images.

#### RGB image processing

To reduce the noise of varying ambient light on RGB images, a one-time color difference correction using a 24-color Macbeth card were performed .Then we extract the positions corresponding to the hyperspectral region of interest (ROI). Those positions in the RGB image are cropped and downsampled to ensure consistency.

#### Hyperspectral image processing

**Figure 1:**
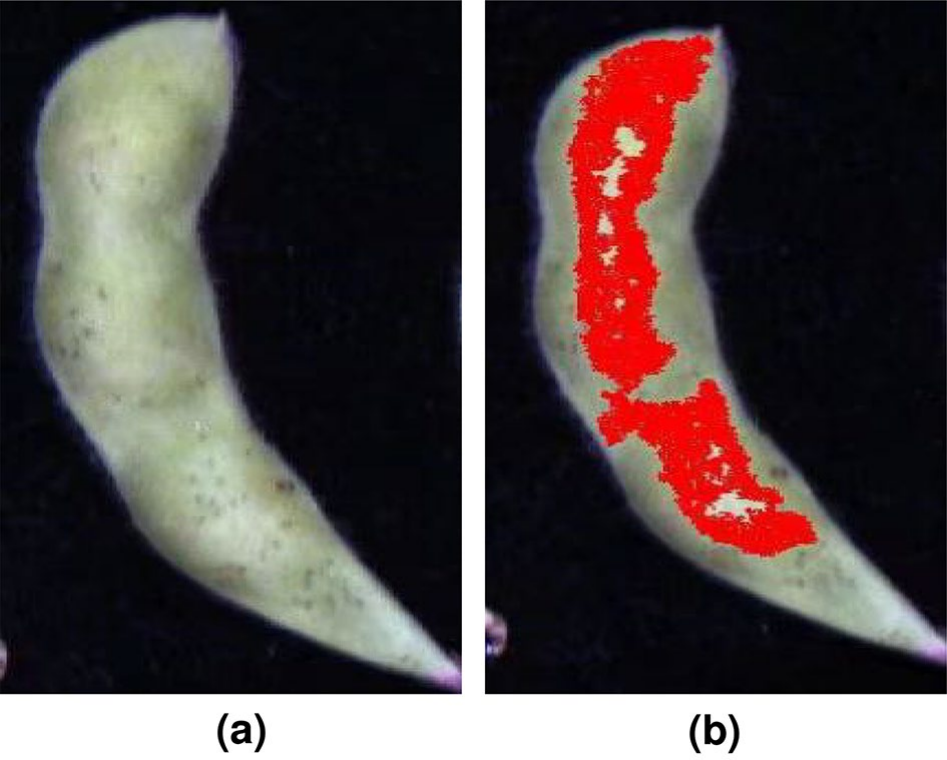
The soybean images and the corresponding ROI. (**a**) The original hyperspectral image. (**b**) The hyperspectral ROI is colored in red.

The ROI was manually selected on the pods with the assistance of ENVI5.3 (ITT, Visual Information Solutions, America)^38–40^, as shown in Fig. 1. Then the average spectrum was calculated. Multiple scattering correction (MSC) was adopted to process the hyperspectral data. It can effectively eliminate the spectral differences^41^. MSC is a commonly used algorithm for hyperspectral data preprocessing. It can effectively eliminate spectral differences due to different scattering levels, and enhance the correlation between spectra and data. MSC corrects the baseline translation and offsets phenomena of spectral data. The detailed steps are as follows.

1. It first derives the average value of all spectral data as the “ideal spectrum”.
2. It performs a linear regression between the spectra of each sample and the ideal spectrum, and solve the least-squares problem to obtain the baseline shift and offset of each sample.
3. It calibrates the spectra of each sample by subtracting the baseline shift and dividing by the offset to derive the corrected spectra.

#### Data fusion model

Combining RGB images with hyperspectral ones in the same model can significantly improve the estimation performance. The RGB images were two-dimensional while the processed hyperspectral data were one-dimensional. The size of the hyperspectral data was 1*462. In accordance with the deep learning model’s prerequisite, it was imperative that the dimensions of both the hyperspectral data and the image data align with each other. Therefore, the hyperspectral data was reconstruct to two dimensions (22$\times$21). For the RGB image data, the entire areas of the soybean pods were extracted and aligned with the hyperspectral data, i.e., the shape of the image data after downsampling was 22$\times$21$\times$3. Then we merged the single-channel hyperspectral data with the three-channel RGB image. The processed hyperspectral data was concatenate with the RGB image data in the color channel. Therefore, our model had 4 input channels.

The fused data were fed into ResNet-R &H. The fused data was four channels and its size was 22*21*4. The mathematical process for the ResNet-R &H model is as follows.

1 $$W_{{in}} *H_{{in}} *D_{{in}} = W_{r} *H_{r} *D_{r} + W_{h} *H_{h} *D_{h} ,$$

where $W_{in}$,$H_{in}$,$D_{in}$ are the width, height and number of channels of the fused data, respectively. $W_r$ is the width; $H_r$ is the height; and $D_r$ is the number of channels of the RGB image. $W_h$ is the width; $H_h$ is the height; and $D_h$ is the number of channels after reshape of the hyperspectral data.

The core idea of ResNet is the introduction of residual block (residual block), whose mathematical expression is as follows.

2 $$\begin{aligned} x_{l+1} = x_{l} + F(x_{l},w_{l}), \end{aligned}$$

where $x_{l}$ is the input of a layer, $x_{l+1}$ is the input of the next layer, and the function F(x, $w_{i}$) is the residual mapping to be learned, which consists of convolution, normalization, and relu activation functions. For a deeper layer L, its relation to layer l can be expressed as follows:

3 $$\begin{aligned} x_{L} = x_{l} + \sum _{i=l}^{L-1}F(x_{i},W_{i}). \end{aligned}$$

The L layer can be represented by the combination of any l-layer network shallower than it and the sum of the residual parts between them. the inputs of the l+1 layer are the outputs of the l layer as follows:

4 $$\begin{aligned} x_{l+1} = f(y_{l}), \end{aligned}$$

where f(x) is the activation function such as ReLU.The residual block is divided into two parts, which are the direct mapping part and the residual mapping part. The generic representation of the residual network is as follows:

5 $$\begin{aligned} y_{l} = h(x_{l}) + F(x_{l},w_{l}), \end{aligned}$$

where h(x) is the direct mapping part, which represents the direct mapping to the input (e.g., a constant transformation, i.e.,h($x_l$)=$x_l$); and f(x,w) is the residual part, which is the result of the input processed through the direct mapping part and the residual mapping part. The final output of the model in our experiments contains four classes.

The ResNet-R &H contains a conv1 stage, Layer1, Layer2, Layer3, Layer4 modules, a pooling layer, and a linear layer.The conv1 stage comprises of a convolutional layer, along with a batch normalization element. This layer operates with 4 input channels and generates 64 output channels. It employs a kernel size of 3, a stride of 1 for convolution, and is accompanied by a padding of 1. The batch normalization layer is used to accelerate the network’s convergence. Layer1 contains two normal residual modules, Layer2 to Layer4 consists of a downsampled convolutional module and a normal residual module.

The convolutional module contains three Conv2d convolutional layers and has two paths. The first path goes through the first convolutional layer, the Relu activation function, and the second convolutional layer sequentially. The second path contains a shortcut convolutional layer. The two paths are summed up and the result goes through the activation function Relu. To ensure shape consistency, the Conv2d convolutional kernel on the shortcut is set to 1. The residual module does not have the convolutional layer at the shortcut and directly adds up the original input. There are 8 blocks of convolutional modules. Then the model has one average pooling layer to deduce the parameters. It can improve the model’s accuracy and stability while reducing overfitting. The final output has four classes, representing four freshness categories.

## Results

### Physicochemical characteristics of VS

**Figure 2:**
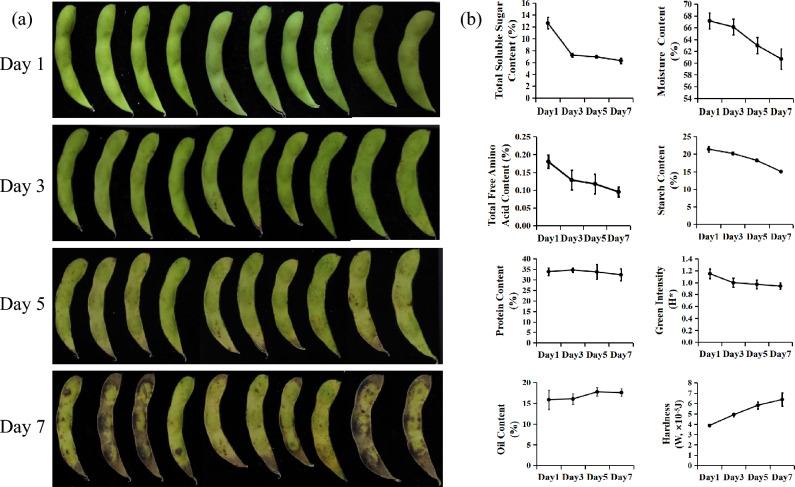
Changes in appearance and nutrients of vegetable soybean with different freshness. (**a**) represents photos of VS pods on the first-, third-, fifth- and seventh-day after storage in the artificial climate chamber, respectively; (**b**) represents line chart of eight physicochemical traits (total soluble sugar, moisture, total free amino acid, starch, protein, oil, green intensity, hardness) change with storage time series.

The freshness of VS decreases as the storage time increases. From an external perspective (Fig. 2a), the pod exhibits a vibrant green color and the surface is smooth and spotless when stored for one day. By the third day, the green intensity and vividness of the pod color began to decline slightly, and a yellowish hue starts to emerge from the base of the pod near the stalk. By the fifth day, this yellowing spreads throughout the entire pod, with brown spots appearing at its stem end. Upon reaching the seveth day, not only does the yellow color intensify but also do these brown spots enlarge and spread across the entire pod, rendering it unsuitable for sale and devoid of market value. The appearance characteristics of the samples showed regular changes with the decrease of freshness. The freshness is negatively correlated with the yellowing color and rust spot areas of the pods. In reality, experienced experts or consumers also measure freshness by observing these changes with their eyes. To give a clearer view of the details of the vegetable soybeans after they decay, we show the pictures of the vegetable soybeans from Day1 and Day7 in Supplement Figure S1.

Besides appearance, physicochemical characteristics of VS are also of great importance to freshness evaluation. Therefore, eight physicochemical traits (total soluble sugar, moisture, total free amino acid, starch, protein, oil, green intensity, hardness) that have been reported to be related to freshness are determined on the first, third, fifth, and seventh days after harvest (Fig. 2b and Supplement Table S1). With the extension of storage time, except for oil and hardness, the other six traits of VS show a downward trend. From the first day to the third day, the soluble sugar content dropped sharply from 12.64 to 7.22%, with a decrease rate of 42.88%. After the third day, the soluble sugar content slowly decreased. On the seventh day, the soluble sugar content is 6.30%, with a decrease rate of 12.68% compared to the third day. From the first day to the seventh day, the total soluble sugar decreased by 50.13%. The changes of total free amino acid content and green intensity are similar to those of soluble sugar content, which decrease rapidly from the first day to the third day and then decreased slowly. From the first day to the seventh day, the total free amino acid content decreased from 0.18 to 0.10%, with a decrease rate of 47.24%, which is slightly smaller than the decrease rate of total soluble sugar (50.13%). From the first day to the third day, the green intensity decreases from 1.15 to 1.00, and on the fifth and seventh days, it decreases to 0.97 and 0.94, respectively. High moisture content is one of the most obvious characteristics of VS as well as other vegetables or fruits. Water is the most abundant component of VS and make up more than 60% of the seed’s wet weight. The moisture content decreases from 67.16% on the first day to 60.70% on the seventh day, with a slow decrease from the first day to the third day and a rapid decrease from the third day to the seventh day. The starch content shows an approximately linear decrease process, from 21.43% on the first day to 15.04% on the seventh day, with a decline rate of 29.80%. The process of protein content change is quite unique. From the first day to the third day, the content slightly increases and then continues to decrease. However, the overall change is not obvious. Among the two increased characters, the hardness increased greatly, from 3.88 ×10^−5^ J to 6.39 ×10^−5^ J , with an increase rate of 64.74%, and the process of change is approximately linear. Oil content increase from 15.88 to 17.60%. In this process, the change from the third day to the fifth day is relatively obvious, while the other time periods have small changes.

### Evaluation metrics

Different measures, including accuracy, precision, recall, and others, were employed for evaluating the efficacy of the model. The confusion matrix was also commonly employed. The main evaluation metrics are as follows:

- True positive (TP): In cases wherein the actual value of the sample is positive, and the projected outcome of the model corresponds positively as well.
- False positive (FP): In cases wherein the actual value of the sample is negative, yet the projected outcome of the model is positive.
- True negative (TN): In cases wherein the actual value of the sample is negative, and the projected outcome of the model corresponds negatively as well.
- False negative (FN): In cases wherein the actual value of the sample is positive, yet the projected outcome of the model is negative.

Accordingly, we employ the following metrics.

- Accuracy: It is the percentage of positive and negative cases correctly predicted.
  6 $$\begin{aligned} Accuracy = \frac{(TP + TN)}{TP + TN + FP + FN} \end{aligned}$$
- Recall: it is the percentage of actual positive cases that are correctly predicted to be positive.
  7 $$\begin{aligned} Recall = \frac{TP}{(TP + FN)} \end{aligned}$$
- Precision: it is the percentage of positive cases predicted by the model as positive.
  8 $$\begin{aligned} Precision = \frac{TP}{TP + FP} \end{aligned}$$
- F1-Score: it is the summed average of the precision and recall scores.
  9 $$\begin{aligned} F1 = 2 * \frac{Precision * Recall}{Precision + Recall} \end{aligned}$$

The results were presented by a confusion matrix. The X axis was the prediction of the model and the Y axis was the number of true labels of the data.

In this work, the technique proposed in this study was compared with four widely used machine learning models as follows.

- Decision tree In machine learning, the decision tree^42^ finds fundamental application for both classification and regression purposes. It has proven to be highly effective in solving classification problems and is widely used in practice. The decision tree operates by selecting the most relevant features in the data, dividing the training samples based on these features, and then recursively repeating this process. The selection of features in the decision tree is based on two criteria: information entropy and information gain. The features with higher information gain were selected as the basis for dividing the data.
- Random forest
  A random forest is a collection of decision trees that combines numerous decisions into a single outcome^43^. This algorithm runs by reconstructing multiple decision trees during the training phase, and it blongs to ensemble learning. Ensemble learning was employed to predict a single outcome by erecting a composite of numerous models. It functions by generating various classifiers, each of which learns and produces predictions autonomously, and then consolidating the ultimate prediction.
- Adaboost
  Boosting, also known as augmented learning, is a crucial technique for integrated learning. It can transform weak learners, which have prediction accuracy only slightly better than random guesses, into strong learners with high prediction accuracy. The AdaBoost algorithm is equivalent to a forward staged additive modeling algorithm that minimizes the loss of new indices used for multi-class classification^44^.
- KNN
  The K-Nearest Neighbor (KNN) classification approach quantifies the gap separating unidentified samples from their established counterparts by referencing known samples spanning various categories^45^. Within this process, the algorithm singles out K known samples, those closest in proximity to the unidentified specimen. Subsequently, adhering to the guideline of minority voting, the categorization of the unidentified samples aligns with the class of the K nearest instances spanning the most extensive array of categories.

### Evaluation results

The current study has partitioned the processed VS samples and their associated images into two distinct sets, the training set was 70% and the test set was 30%. Notably, the selection of samples for each set was executed randomly. Moreover, the distribution of each category remains consistent in both sets, satisfying the principle of proportionality. The algorithms were run on a system equipped with an Intel Xeon Gold 5218 CPU, NVIDIA Tesla V100 GPU, and Ubuntu 18.04 operating system. The classification model was trained using Python (version 3.6.8) and PyTorch (version 1.7.1).

The method was evaluated through three datasets: the hyperspectral dataset, the RGB image dataset, and the fused dataset. Each dataset consists of four classes of VS with varying freshness levels. The datasets contain 462 bands of hyperspectral data, which was reprocessed into a single channel of $22\times 21$ data. The colored images were also resized to $22\times 21$ with a total number of 416.

**Figure 3:**
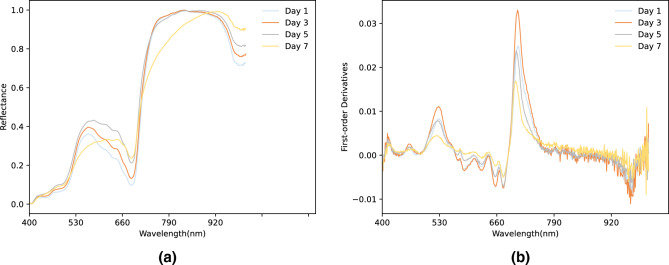
Normalized spectral values and first-order derivatives corresponding to different bands of vegetable soybeans at four freshness levels. (**a**) Normalized spectral. (**b**) First order derivative.

Firstly, a waveband analysis on VS of varying freshness levels was performed. The hyperspectral values of each VS were then averaged and normalized. Fig. 3a illustrates the normalized spectral values corresponding to different bands of VS at four freshness levels. Fig. 3b shows the first-order derivatives corresponding to different bands of VS at the same freshness levels. As shown in Fig. 3a, there is one peak at 562 nm and one trough at 688 nm. The results of the first-order derivative analysis in Fig. 3b demonstrate that the spectral reflection separation between the types and levels of VS freshness mainly occurs within the 494 nm to 681 nm and 695 nm to 764 nm range^46^. The spectral values within this range vary significantly, indicating that the spectrum in this band is strongly correlated to VS freshness.To better indicate the location of the peaks and valleys of the wave band in Fig. 3a and the reflectance separation band region in Fig. 3b, the 500 nm–710 nm region of Fig. 3a was zoomed (Supplement Figure S2). The 420nm-700nm region of Fig. 3b (Supplement Figure S3), and the 650nm-770nm region (Supplement Figure S4) are also amplified.

Then, VS samples were classified using machine learning techniques and our deep learning method based on RGB image data and hyperspectral data separately. Table 1 presents the accuracy, average precision, average recall, and average F1 score metrics for four machine learning models and deep learning models trained with hyperspectral data, image, and fused data, respectively. The results indicate that the highest testing accuracy achieved by machine learning models is 87.2%, while the deep learning method achieves the highest accuracy of 97.6% among all the types of data. And the values of precision, recall, and F1-score are all higher than those of traditional machine learning models. This demonstrates that the model developed in this study significantly enhances the classification performance. Due to the small number of samples and high accuracy, we use Wilson’s method to derive the confidence intervals. We calculate the confidence intervals for the 125 test samples with 97.6% classification accuracy at 95% confidence level and 99% confidence level, respectively. The results are 93.18%–99.18% and 93.15%–99.50%. This demonstrates the reliability of our method and show enough statistical significance.

**Table 1:** Evaluation metrics of the classification effects of five classification models on VS freshness based on RGB image data, hyperspectral data, and fusion data.

	Decision tree (%)	KNN (%)	Random forest (%)	AdaBoost (%)	ResNet-R &H (%)
Accuracy					
RGB image	77.6	80.0	84.0	77.6	90.4
Hyperspectral	79.2	86.4	82.4	83.2	93.6
Fusion data	84.0	87.2	84.8	87.2	97.6
Precision					
RGB image	76.0	83.6	83.8	78.6	91.3
Hyperspectral	79.2	86.7	82.1	83.2	93.5
Fusion data	87.0	87.0	84.9	86.9	98.0
Recall					
RGB image	75.9	81.8	84.9	78.1	91.5
Hyperspectral	78.1	86.3	82.6	83.1	93.5
Fusion data	87.1	86.8	85.2	87.4	97.5
F1-Score					
RGB image	75.4	79.7	84.0	77.6	90.5
Hyperspectral	78.3	86.4	82.3	83.0	93.5
Fusion data	87.0	86.9	85.1	87.0	97.8

The hyperspectral data used for model training is based on the full bands.

Table 2 shows the processing time of 125 samples from the test set using different methods. When the data sources are RGB images and hyperspectral images, the time required by the proposed ResNet-R &H method in this study is 13.80 ms and 10.18 ms, respectively, which are higher than the remaining four machine learning models. When the data source is fused data, its required time is 15.23ms, which is only 1.43–5.05 ms higher than that based on the single source data. And the ResNet-R &H requires more inference time than that of DecisionTree/KNN/RandomForest based on the fusion data, the time gap required to process 125 samples is only 4.38–11.29 ms, which is generally acceptable in real-world applications. What’s more, the computation time of the ResNet-R &H model is even much lower than that of AdaBoost.

**Table 2:** Time required for inference test set of 125 samples under different methods.

	Decision tree	KNN	Random forest	AdaBoost	ResNet-R &H
Time(ms)					
RGB image	0.37	7.31	7.74	10.16	13.80
Hyperspectral	0.27	6.09	7.69	3.76	10.18
Fusion data	3.94	9.56	10.85	38.08	15.23

**Figure 4:**
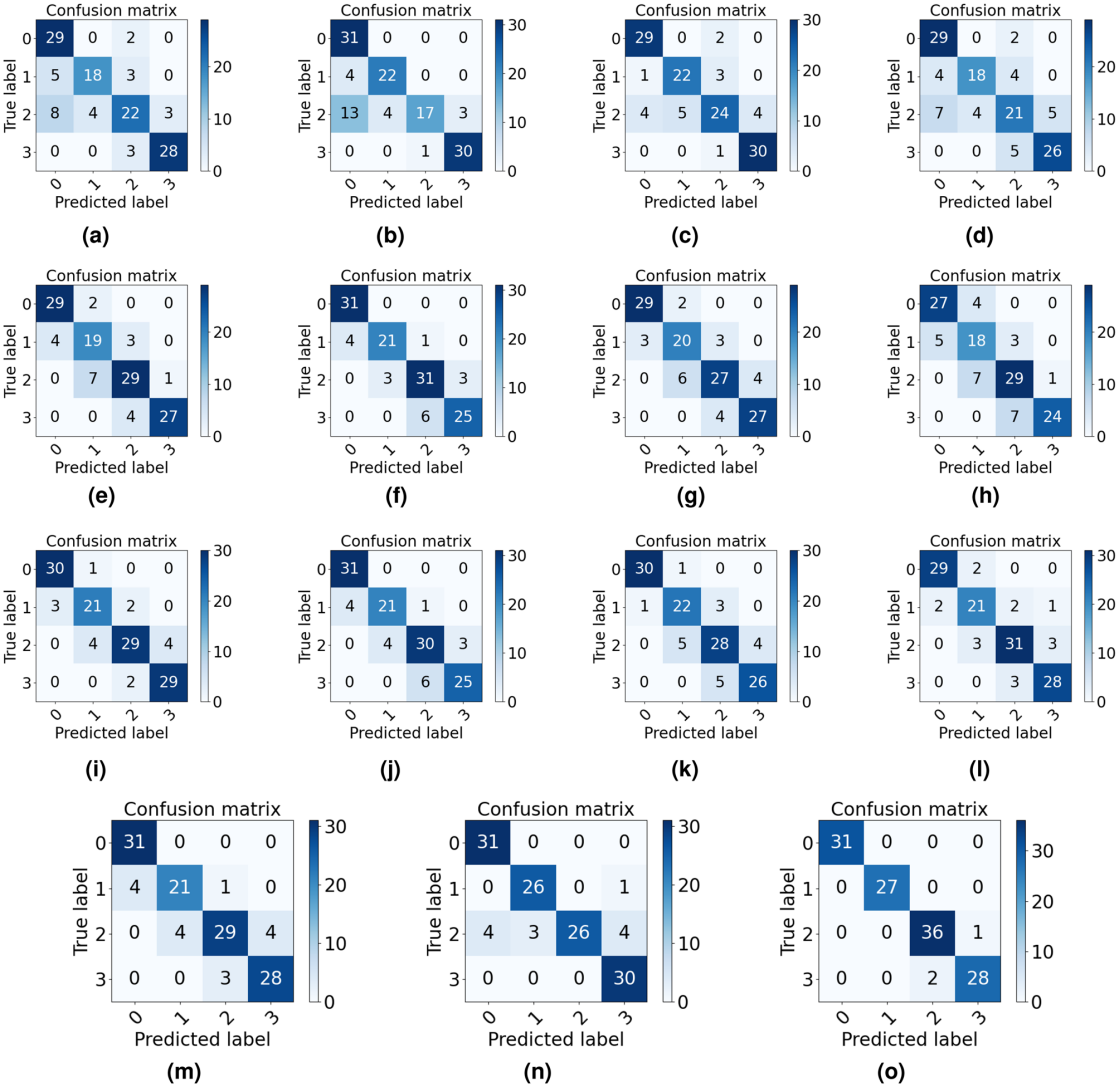
Classification confusion matrix. (a-i): Confusion matrix of four machine learning methods (AdaBoost, KNN, Random forest, and Decision tree) based on RGB images (**a**−**d**), hyperspectral images (**e**–**h**), as well as fusion data (**i**–**l**). (**m**–**o**): Confusion matrix of deep learning method based on RGB image, hyperspectral image and fusion data, respectively. The axis labels 0,1,2,3 represent Day1,Day3, Day5,Day7, respectively. The numbers in the confusion matrix represent the corresponding number of samples, and the total sample size is 125.

Figure 4 shows the confusion matrix of VS freshness classification under four machine learning methods and one deep learning method. Comparing Fig. 4a–d and e–h, it is clear that these models trained solely on RGB or hyperspectral data have considerably lower classification. This suggests that relying upon a single data source suffers from incomplete representation of certain essential characteristics. Therefore, using fused data has led to a marked improvement in classification accuracy, precision, recall, and F1 score.

### Band ablation analysis

**Figure 5:**
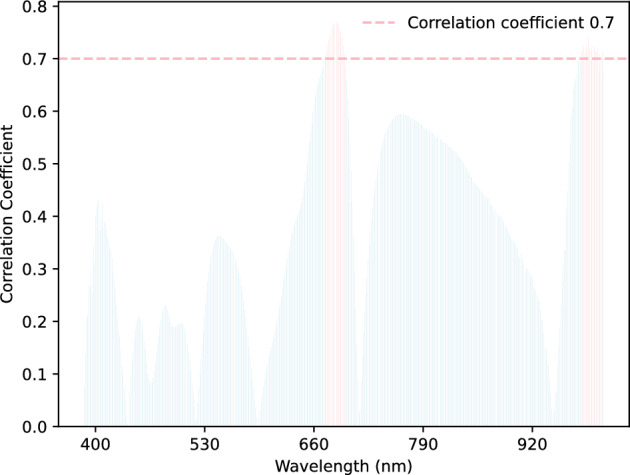
The correlation between the hyperspectral reflectance of vegetable soybeans and corresponding freshness. The bands with a correlation coefficient greater than 0.7 are marked in pink.

**Table 3:** Evaluation metrics of the classification effects of five classification models on VS freshness based on hyperspectral data and fusion data.

	Decision tree (%)	KNN (%)	Random forest (%)	AdaBoost (%)	ResNet-R &H (%)
Accuracy					
hyperspectral	75.2	66.4	76.8	82.4	88.0
fusion data	83.2	73.6	92.0	92.8	92.8
Precision					
hyperspectral	75.6	68.3	78.1	82.7	87.8
fusion data	83.2	73.5	92.0	92.6	92.8
Recall					
hyperspectral	75.5	67.1	76.9	82.4	87.5
fusion data	83.9	74.5	92.3	93.1	93.5
F1-Score					
hyperspectral	75.1	64.2	76.8	82.3	87.5
fusion data	83.0	73.6	92.1	92.1	93.0

The models were trained from 32 bands of data with correlation coefficient greater than 0.7, and the rest of the bands were set to 0.

The characteristic bands most closely related to freshness were identified. Instead of utilizing the full-band data, feature band selection on the hyperspectral bands was performed^47–49^.In Fig. 5, the correlation analysis was employed to investigate the relationship between various wavelengths of data and their corresponding freshness categories. The correlation between bands and freshness was analyzed using the distance correlation coefficient^50,51^. It was calculated by dividing the distance covariance of two random variables with the product of their distance standard deviations. The detailed equation is shown as follows.

10 $$\begin{aligned} dCor(X,Y) = \frac{dCov(X,Y)}{\sqrt{dVar(X)dVar(Y)}}. \end{aligned}$$

A stronger correlation was indicated by a correlation coefficient closer to 1. The experimental results show a series of highly correlated wavelengths around 670 nm and 980 nm. The distribution of selected wavelengths closely resembles the outcomes of the first-order derivative analysis, indicating that peaks and valleys at wavelengths correspond to higher correlations.

We selected 32 bands (673 nm–695 nm and 979 nm–1003 nm) with correlation coefficients greater than 0.7 to train models. All the other bands were set to zero. The results are shown in Table 3. It is observed that the ResNet-R &H model achieves the highest accuracy in general. The test accuracy obtained by the AdaBoost method is nearly equal to the full-band accuracy (462). The RandomForest method is about 5.6% lower than the fullband model. KNN has a 20% drop in accuracy compared to the full band.

According to results of the experiment, the RandForest and AdaBoost methods have achieved 92.0% and 92.8% accuracy, respectively. Which are higher than those trained on the full-band fused data, with values of 84.8% and 87.2% (Table 1 and Table 3. The feature selection can select the feature bands that are most closely related to the freshness, thus reducing the hardware requirement. Moreover, the average time required to train the network reduced from 3.634 to 3.465 s per epoch.

## Discussion

Through visual and physicochemical indicators (Fig. 2), differences can be observed between samples at different freshness levels. The classification of vegetable freshness based on sensory evaluation and physical and chemical characteristics detection is a time-consuming and laborious task, and the results are difficult to reproduce. Therefore, there is a growing interest in non-contact technologies utilizing image and spectral analysis to achieve rapid and automated classification of vegetable freshness. At present, hyperspectral technology has been more and more widely used in the study of vegetable and fruit freshness. The research results of Polder et al.^52^show that hyperspectral images have more advantages than ordinary RGB images in identifying the maturity of tomatoes, and the recognition and classification error of single pixel decreases from 51% to 19%. However, the efficiency of freshness classification is not high enough due to ignoring the external characteristics of the sample^53^. The accuracy of classification results may be compromised by relying solely on a single source of data. And we experimentally prove this corollary in our study(Table 1). It is imperative to consider the fusion of data from multiple sources to improve classification accuracy. For example, there exist blurring distinctions between Day 1 and Day 3 observed from photos (Fig. 2a), while significant changes occur in the levels of chemicals like soluble sugars and free amino acids (Fig. 2b), which can be rapidly and sensitively detected with hyperspectral techniques (Fig. 3). In contrast, the contents of total soluble sugar, protein, and oil showed no difference between Day 5 and Day 7 (Fig. 2b), but the samples can be preliminarily distinguished visually (Fig. 2a). And this subjective visual distinction of the sample’s appearance changes can be more accurately and efficiently performed through RGB images. Therefore, the above results further illustrate that fusion data from both RGB and hyperspectral sources can effectively improve the accuracy and precision of freshness classification when compared to relying on a single data source (Table 1). Actually, this inference is confirmed in the subsequent experimental results of this paper. From the confusion matrix in Fig. 4m-o and Supplement Figure S5, it is demonstrated that the deep learning classification method based on RGB images misclassified 4 samples belonging to Day1 as Day3 (Fig. 4m). When the data source changes to hyperspectral (Fig. 4n) or fused data (Fig. 4o), the number of misclassifications is reduced to 0. On the contrary, there is a significant difference in the appearance of the samples on Day 3 and Day 7, which can be correctly distinguished through RGB images (Fig. 4m). However, there is still one sample misclassified based on hyperspectral images (Fig. 4n). Above all, based on the fusion data can effectively reduce the occurrence of the above mentioned misclassification situation (Fig. 4o), which highlighting the strength of the proposed model.

Hyperspectral imaging techniques enable the simultaneous acquisition of images at different wavelengths. In this work, the hyperspectral data have 462 wave bands. Due to the small sampling interval of imaging spectrometers, adjacent bands have high correlations and suffer from information redundancy. An RGB image is an array of color pixels of size M×N×3 (M×N pixel points, 3 channels), and each color pixel point is a composition of red, green, and blue components. The RGB camera decomposes the spectrum into three broad bands to capture images with superior spatial resolution but limited spectral resolution. Hyperspectral imaging techniques can simultaneously acquire images of different wavelengths in the same scene. However, the existing hyperspectral imaging equipment has low spatial resolution^54–56^. The fusion of high spatial-resolution RGB images with low-resolution hyperspectral images can improve classification accuracy^57^.

This work proposes a deep learning-based method to classify the freshness of VS based on RGB and hyperspectral image data. It combines hyperspectral data and image data to effectively distinguish the VS freshness. As shown in Table 1, the classification accuracy of VS freshness is significantly improved.

In the data preprocessing stage, the input image is downsampled. The downsampling processed image reduces the pixel points of the RGB image, thus reducing the model input complexity. The reduction of input data can shorten the training and inference time. Overall, the classification accuracy of the data fusion-based approach can be significantly improved with a quite slightly increased of inference time.

A feature selection model is proposed to select the most relevant bands. The correlation coefficient calculation is used, which requires limited resources. By reducing the input bands number from 462 to 32, performance for most methods decreases, except for RandomForest and AdaBoost. Their accuracies remain at 92.0% and 92.8% with fused input, respectively. For incomplete data sets, Random Forest can handle missing values and incomplete features. When building decision trees, Random Forest uses a random subset of features for training, so that even if some features are missing, other features can be used for prediction. The AdaBoost algorithm can handle incomplete data sets because it is an iterative algorithm that gradually adjusts the model to fit the missing data. In each iteration, AdaBoost adjusts the sample weights to focus more attention on the misclassified samples, thus improving the adaptation to the missing data. Such experimental results show that the feature selection method reduces the training time with less impact on the classification accuracy. In addition, our method offers the possibility to propose training algorithms with reduced training samples.

The method proposed in this study can provide a theoretical reference for classifying the freshness of other kinds of fruits and vegetables to improve classification accuracy and reduce human error and variability. The appearance and nutrient content of different vegetables or fruits are very different. With the decrease of maturity, the change trend of physical and chemical characteristics is also very different from that of vegetable soybeans. Therefore, our proposed model may only be applicable to the freshness classification of vegetable soybeans. In the later stage, the type and number of experimental samples can be expanded to improve the discrimination accuracy and generalization of the model.

## Conclusions

In this study, we propose a novel classification model called ResNet-R &H, which is based on the residual networks (ResNet) structure and incorporates the fusion data of RGB and hyperspectral images. ResNet-R &H is significant in providing a unique, efficient, and more accurate classification approach in evaluating the freshness of vegetable soybean.

### Supplementary Information

Supplementary Information 1.

Supplementary Information 1.

Supplementary Information 1.

Supplementary Information 1.

Supplementary Information 1.

Supplementary Information 1.

## Data Availability

Our dataset and code will be publicly available at https://github.com/HZSUZJ/DLDF.
