# Supplementary figures and images for: ResNet incorporating the fusion data of RGB & hyperspectral images improves classification accuracy of vegetable soybean freshness
Source: Sci Rep. 2024 Jan 31;14:2568. doi: 10.1038/s41598-024-51668-6 (PMC11224382; doi:10.1038/s41598-024-51668-6)

Figure S1.Enlarged detail view of Day1 and Day7 vegetable soybean.

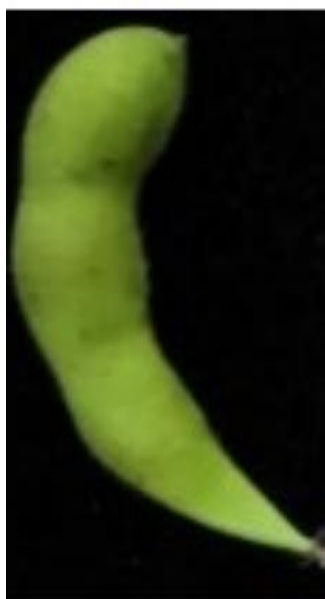

Day1

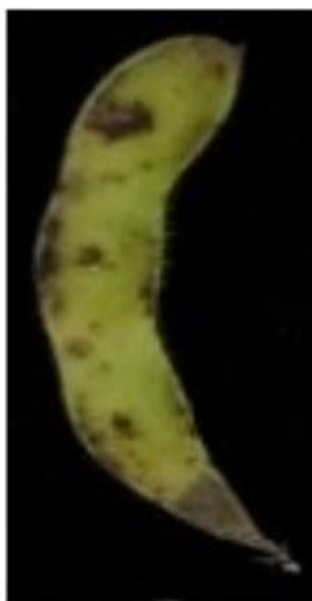

Day7

Supplement: Supplementary file 1 — Supplementary Information 1. [file 41598_2024_51668_MOESM1_ESM.pdf]

Figure S2. Magnified view of the 500 nm-710 nm region in Figure 3(a)

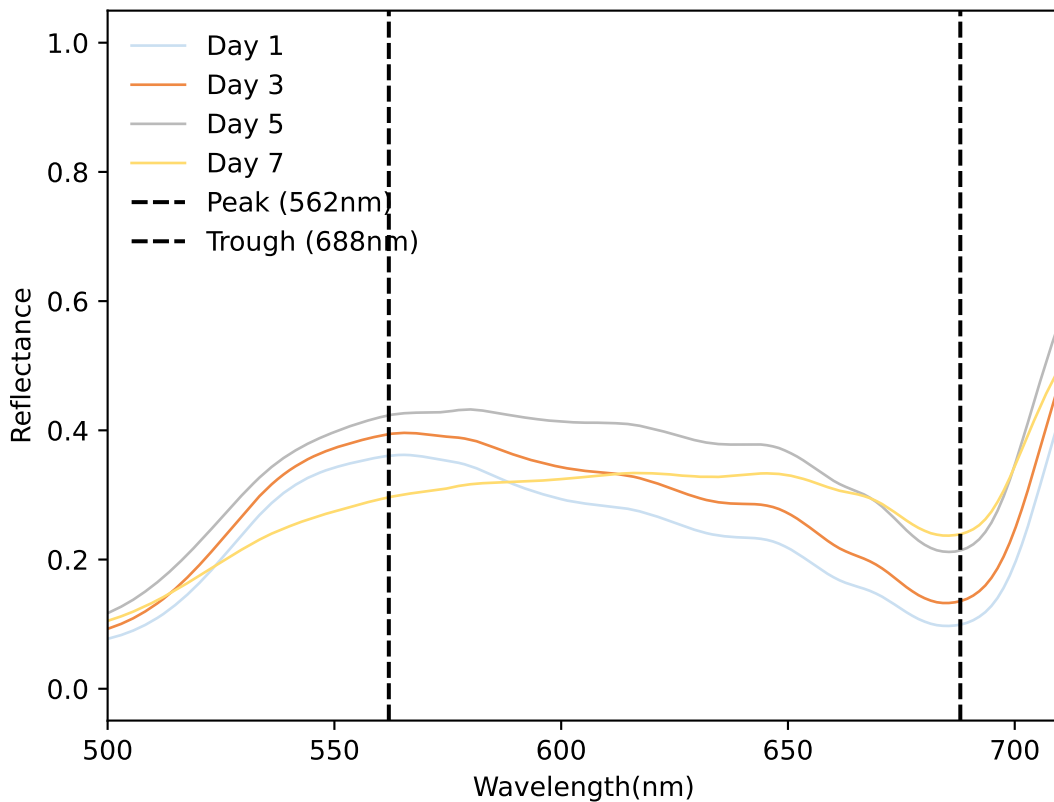

Supplement: Supplementary file 2 — Supplementary Information 1. [file 41598_2024_51668_MOESM2_ESM.pdf]

Figure S3. Magnified view of the 420 nm-700 nm region in Figure 3(b)

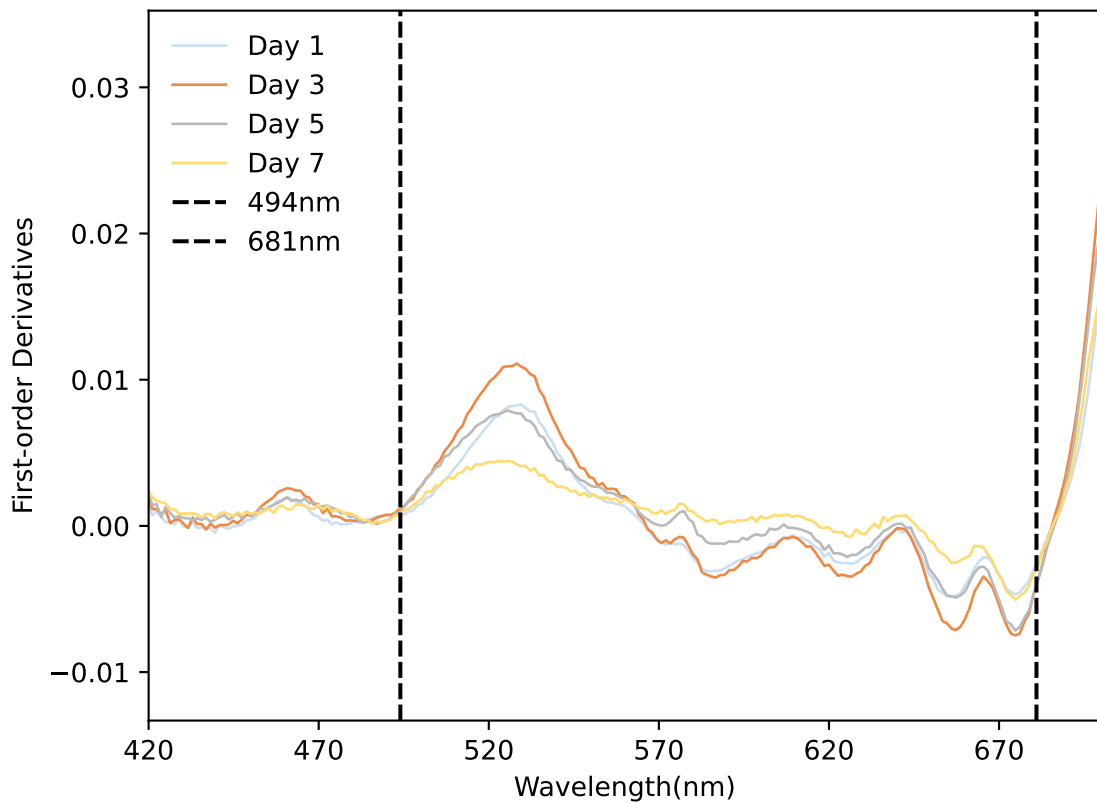

Supplement: Supplementary file 3 — Supplementary Information 1. [file 41598_2024_51668_MOESM3_ESM.pdf]

Figure S4. Magnified view of the 650 nm-770 nm region in Figure 3(b)

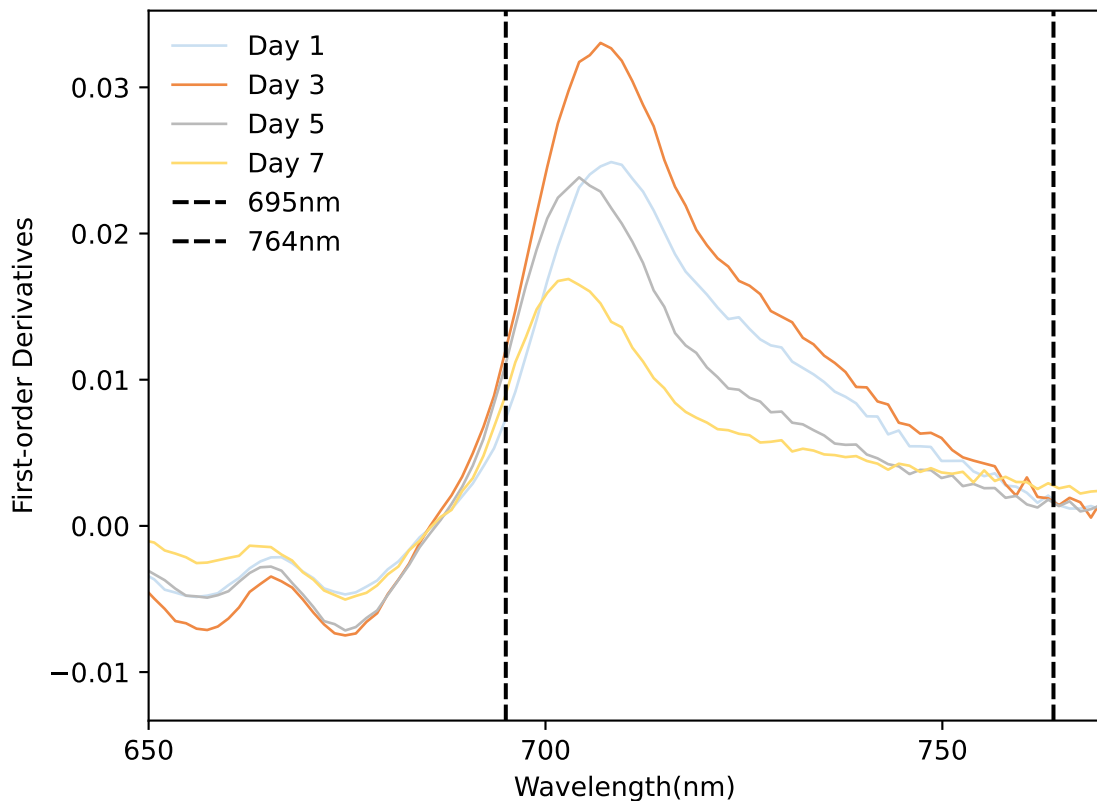

Supplement: Supplementary file 4 — Supplementary Information 1. [file 41598_2024_51668_MOESM4_ESM.pdf]

Figure S5.Enlarged detail view of Day1 and Day3 vegetable soybean.

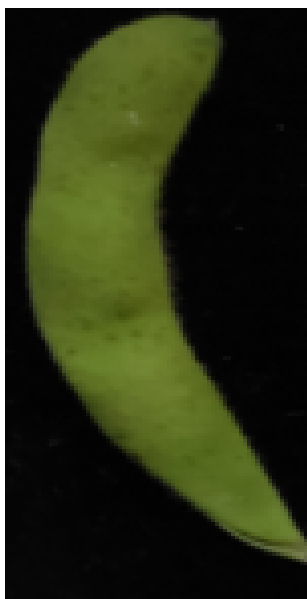

Day1

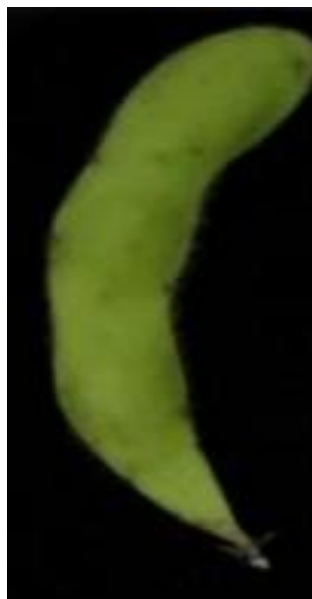

Day3

Supplement: Supplementary file 5 — Supplementary Information 1. [file 41598_2024_51668_MOESM5_ESM.pdf]
