# Supplementary material for: ResNet incorporating the fusion data of RGB & hyperspectral images improves classification accuracy of vegetable soybean freshness
Source: Sci Rep. 2024 Jan 31;14:2568. doi: 10.1038/s41598-024-51668-6 (PMC11224382; doi:10.1038/s41598-024-51668-6)
Supplement: Supplementary file 6 — Supplementary Information 1. [file 41598_2024_51668_MOESM6_ESM.pdf]

Table S1. Average and standard deviation (SD) of eight physicochemical traits change with storage time series.

Traits	Day 1		Day 3		Day 5		Day 7	
	Average	SD	Average	SD	Average	SD	Average	SD
Total Soluble Sugar (%, dry weight)	12.64	0.98	7.22	0.32	6.93	0.20	6.30	0.44
Moisture (%, wet weight)	67.16	1.37	66.13	1.34	62.99	1.39	60.70	1.73
Total free amino acid (%, wet weight)	0.18	0.02	0.13	0.03	0.12	0.03	0.10	0.01
Starch (%, dry weight)	21.43	0.78	20.22	0.36	18.22	0.29	15.04	0.25
Protein (%, dry weight)	33.90	1.83	34.67	1.09	33.74	3.55	32.39	2.83
Oil (%, dry weight)	15.88	2.28	16.06	1.30	17.78	1.00	17.60	0.93
Hardness ($\times 10^{-5}$J)	3.88	0.11	4.92	0.16	5.83	0.33	6.39	0.62
Green intensity	1.15	0.08	1.00	0.07	0.97	0.07	0.94	0.05
